# Evaluation of osteogenic potential of demineralized dentin matrix hydrogel for bone formation

**DOI:** 10.1186/s12903-023-02928-w

**Published:** 2023-04-28

**Authors:** Nessma Sultan, Soher Nagi Jayash

**Affiliations:** 1 Department of Oral Biology, Faculty of Dentistry, Mansoura University, Mansoura, Egypt; 2 Roslin institute, University of Edinburgh, Edinburgh, UK

**Keywords:** Bone marrow mesenchymal stem cells, Demineralized dentin matrix hydrogel, Nano-hydroxyapatite hydrogel

## Abstract

**Objectives:**

Dentin, the bulk material of the tooth, resemble the bone’s chemical composition and is considered a valuable bone substitute. In the current study, we assessed the cytotoxicity and osteogenic potential of demineralized dentin matrix (DDM) in comparison to HA nanoparticles (n-HA) on bone marrow mesenchymal stem cells (BMMSCs) using a hydrogel formulation.

**Materials and methods:**

Human extracted teeth were minced into particles and treated via chemical demineralization using ethylene diamine tetra-acetic acid solution (EDTA) to produce DDM particles. DDM and n-HA particles were added to the sodium alginate then, the combination was dripped into a 5% (w/v) calcium chloride solution to obtain DDM hydrogel (DDMH) or nano-hydroxyapatite hydrogel (NHH). The particles were evaluated by dynamic light scattering (DLS) and the hydrogels were evaluated via scanning electron microscope (SEM). BMMSCs were treated with different hydrogel concentrations (25%, 50%, 75% and neat/100%) and cell viability was evaluated using MTT assay after 72 h of culture. Collagen-I (COL-I) gene expression was studied with real-time quantitative polymerase chain reaction (RT-qPCR) after 3 weeks of culture and alkaline phosphatase (ALP) activity was assessed using enzyme-linked immune sorbent assay (ELISA) over 7th, 10th, 14th and 21st days of culture. BMMSCs seeded in a complete culture medium were used as controls. One-way ANOVA was utilized to measure the significant differences in the tested groups.

**Results:**

DLS measurements revealed that DDM and n-HA particles had negative values of zeta potential. SEM micrographs showed a porous microstructure of the tested hydrogels. The viability results revealed that 100% concentrations of either DDMH or NHH were cytotoxic to BMMSCs after 72 h of culture. However, the cytotoxicity of 25% and 50% concentrations of DDMH were not statistically significant compared to the control group. RT-qPCR showed that COL-I gene expression was significantly upregulated in BMMSCs cultured with 50% DDMH compared to all other treated or control groups (*P < 0.01)*. ELISA analysis revealed that ALP level was significantly increased in the groups treated with 50% DDMH compared to 50% NHH after 21 days in culture (*P < 0.001)*.

**Conclusion:**

The injectable hydrogel containing demineralized dentin matrix was successfully formulated. DDMH has a porous structure and has been shown to provide a supporting matrix for the viability and differentiation of BMMSCs. A 50% concentration of DDMH was revealed to be not cytotoxic to BMMSCs and may have a great potential to promote bone formation ability.

## Introduction

Bone remodelling is a dynamic physiological process which is necessary for maintaining the skeleton architecture, which response to mechanical stimuli. Successful bone remodelling necessitates a balance between bone formation and resorption. When such balance undergoes disruption, some disorders developed including osteoporosis [1, 2]. Current bone substitutes fail in the recapitulation of the unique remodelling capacity of bones. Autologous bone is considered the gold standard for bone defect regeneration; however, it is of a limited amount and can cause donor site morbidity. To overcome such limitations, many efforts have continued to develop an ideal bone graft material [3].

Hydroxyapatite (HA) is a ceramic biomaterial, and it is the main mineral component of teeth and bones. Exceptional biocompatible characteristics of nano-hydroxyapatite (n-HA) made it significantly used in bone regeneration and is also used for multipurpose applications in biomedical industries and dental applications. The n-HA-related remineralizing effect is more useful than HA in bone regeneration [4]. It stimulates the proliferation of mesenchymal cells, but due to its low crystallinity and larger surface area, it is more soluble and more likely to interact with the environment. This may consequently cause the surface release of calcium Ca2 + to rise. The environment around the bones and cell proliferation may be severely impacted by the abrupt elevations in Ca2 + concentrations in bodily fluids [5, 6].

Bones and teeth are extremely similar in chemical composition, and both composed of an inorganic HA and organic matrix, comprising mainly collagen and non-collagenous proteins. The teeth minerals include five types of biologic calcium phosphates (HA, tricalcium phosphate, octacalcium phosphate, amorphous calcium phosphate, and dicalcium phosphate dehydrate). The five types of calcium phosphates produce a good bone remodelling after implantation [7, 8]. The apatite in bone shows a low-crystalline structure, and its particle size ranges from 10 to 100 nm. But, when made by sintering at a high temperature, HA has a high-crystalline structure, resulting in a larger size than apatite in the bone. These changes in crystallinity along with the large particle size makes biodegradation extremely difficult as bone turnover is significantly reduced, and the HA cannot be degraded by osteoclasts [7–9].

Odontoblasts are specialized cells responsible for dentin formation. The process of odontoblast differentiation is well controlled by a plethora of bioactive molecules including transforming growth factors, epidermal growth factors, fibroblast growth factors, bone morphogenic proteins (BMPs) and collagen type I (COL-I). After odontoblastic differentiation and dentin formation, these molecules become entrapped within the matrix [10]. Thus dentin matrix (DM) contains many natural proteins and bioactive compounds, which have wide application prospects in bone and dentin regeneration [7–12]. The osteoinduction capacity of dentin makes it an appropriate candidate for bone regeneration and raises the possibilities of teeth bio-recycling which is considered a waste product in the dental clinic [13, 14]. Based on previous reports, it is believed that the teeth can be utilized for developing bone grafts with a healing capacity comparable to autogenous bone [11, 12].

The potential for the development of hydrogels for maxillofacial bone repair is enormous. Researchers have investigated adding various elements to hydrogels to improve their biological and mechanical properties [15, 16]. Alginate hydrogel is commonly utilized for 3D culture and osteoinduction of bone marrow mesenchymal stem cells (BMMSCs) as it can combine with variable osteogenic induction cues [17]. Hence, the current study aimed to evaluate the cell viability and osteogenic potential of sodium alginate composite hydrogel loaded with either demineralized dentin matrix (DDM) or n-HA on BMMSCs. Osteogenic markers such as alkaline phosphatase (ALP) activity and specific gene expression (COL-I) were evaluated. The null hypothesis tested was that no difference could be found in the osteogenic potential of either DDMH or NHH on BMMSCs in vitro.

## Materials and methods

The collection and storage of the teeth, animal handling and all the experimental protocols were approved by the Faculty of Dentistry, Mansoura university ethical committee with number; A23060722. The rats used to isolate BMMSCs were white albino rats supplied by mansoura experimental research centre.

### Isolation and characterization of BMMSCs

BMMSCs were isolated from 5 to 6 weeks white albino rat’s femur. Sectioned bones were cleaned from all connective tissues and were collected on ice in 10 ml isolation medium (IM). IM medium contained basic medium Dulbecco modified essential medium/F12 (DMEM/F12) with L-glutamine (Life science, UK) supplemented with 20% fetal bovine serum (FBS, Life science, UK), penicillin (100 µg/mL) and streptomycin (100 µg/mL) (Biowest, USA). One-hundred-millimeter petri dish (Sterilin, Fisher scientific, UK) was aseptically set up for processing of each bone in a biohazard laminar flow hood. BMMSCs were collected by flushing each long bone with 10 ml of complete culture medium, containing DMEM/F12 with 20% FBS, 1% antibiotic-antimycotic, and filtered through a 70 *µ*m nylon cell strainer into T-75 tissue culture flasks (Greiner Bio-One International, Kremsmünster, Austria). Cells were incubated at 37 °C (5% CO_2_), and nonadherent cells were removed by change of medium after 24 h. The culture medium was changed every 2–3 days.

#### Characterization

To examine surface molecular expression in BMMSCs, 5 × 10^5^ cells (3rd passage) in 1.5 ml eppendorf tubes were fixed with 4% paraformaldehyde for 15 min and then washed in phosphate buffer saline (PBS) and labelled with primary CD105, CD90, CD34 and CD45 antibodies at room temperature for 1 h. After that, they were labelled with fluorescein isothiocyanate-conjugated secondary antibodies at room temperature in the dark for 45 min. The percentages of cells staining positive for CD90, CD105 and percentages of cells staining negative for CD34, CD45 were assessed using FACS calibre flow cytometry (BD Immunocytometry Systems).

### Characterization of n-HA and DDM particles

The prepared particles were analyzed for their particle size, polydispersity index (PDI), and zeta potentials of n-HA and DDM particles were measured by a dynamic light scattering instrument (Zetasizer Nano ZS90, Malvern, Worcestershire, UK) at a fixed angle of 173° at 25° C. All measurements were obtained in triplicate. All the samples underwent dilution with distilled water before analysis.

### Formulation of demineralized dentin matrix hydrogel (DDMH)

A total of ten (n = 10) caries-free permanent premolar teeth were collected from the outpatient clinic of the Faculty of Dentistry, Mansoura University, Egypt. The collected teeth were extracted for orthodontic reasons. The collection and storage of teeth were subjected to infection control standards approved by the Faculty of Dentistry ethical committee. The enamel and cementum were eliminated by the high-speed handpiece, and the teeth dentin were immersed in isopropyl alcohol for 2 h, and then were kept in a saline solution. The teeth dentin were cleaned with distilled water 3 times using an ultrasonic cleaner (Ultrawave, UK) for 10 min each. The teeth dentin were ground by the grinder (coffee miller) and then were demineralized in a reducing gradient of ethylenediamine tetra acetic acid (EDTA) concentrations as follow: 17 mol/L (5 min), 10 mol/L (5 min), 5 mol/L (10 min). After demineralization, the teeth particles were ultrasonically cleaned in distilled water for 10 min and then were freeze-dried [18].

Initially, 0.125 g of sodium alginate (Sigma-Aldrich, St. Louis, US) was aseptically dispersed in 2.5 ml distilled water and glycerol (as plasticizer) to obtain 5% (w/v) of sodium alginate. Then this solution was filtered using 0.45-µm syringe filters (Thermofisher scientific, UK). After that, 0.125 g of DDM particles were dispersed in the solution with a mass ratio of 1:1. The hydrogel was then produced by dripping the solution into a cross-linking calcium chloride (CaCl_2_) 5% (w/v) solution (Sigma-Aldrich, St. Louis, MO, US). The hydrogel was transferred into a single syringe to obtain a uniform injectable hydrogel mass [19].

#### Formulation of Nano-hydroxyapatite hydrogel (n-HA)

A sodium alginate powder (0.125 g) was aseptically dispersed in 2.5 ml distilled water and glycerol (as plasticizer) at room temperature to form 5% (w/v) of sodium alginate. The solution then was filtered using 0.45-µm syringe filters. After that, this solution was mixed with n-HA powder (nanopowder, < 200 nm particle size (BET), > 97%, synthetic, Sigma-Aldrich, St. Louis, MO, US) to achieve a final concentration of 5 mg/mL [20]. After homogeneity, the mixture was dripped into a sterile cross-linking CaCl_2_ 5% (w/v) solution.

### Preparation of different hydrogel concentrations

A dose-response relationship was assessed by diluting the tested hydrogels with complete culture medium (DMEM/F12 + 10% FBS) to achieve 4 concentrations of each hydrogel (25%, 50%, 75% and neat or 100%). These different concentrations were cultured with BMMSCs to test their cytotoxicity by using 3-(4,5-dimethylthiazol-2-yl)-2,5-diphenyl-2 H-tetrazolium bromide (MTT) assay (MTT Cell Growth Kit; Chemicon, Rosemont, IL). To test the osteogenic potential of the different hydrogel concentrations; COL-I gene expression was assessed using RT-qPCR and ALP activity was assessed using Enzyme-linked immunosorbent assays (ELISAs). Cells seeded in complete culture medium were used as controls.

### Scanning electron microscopy (SEM)

To evaluate the cross-sectional morphology of the formulated hydrogels, SEM (Jeol JSM 6510, Jeol, Peabody, MA) was used. After gold-sputtering, the hydrogels were mounted onto SEM stubs and were examined utilizing an accelerating voltage of 15 kV and magnification 500x.

### Cytotoxicity evaluation

BMMSCs in the 3rd passage were seeded at a density of 4000 cells/cm^2^ (96-well plate) in 200 µL DMEM/F12 supplemented with 10% FBS. After 24 h, the culture medium was replaced by 200 µL of different concentrations of the tested hydrogels (25%, 50%, 75% and neat/100%) and incubated for 72 h. MTT was added at a final concentration of 1 mg/mL for 4 h. After that, the removal of the culture medium containing MTT was done with the addition of 100 µL dimethyl sulfoxide (Thermofisher scientific, UK) to release formazan. The absorbance was measured at 570 nm by a microplate reader (ELx800; Bio-Tek Instruments, Winooski, VT) and also absorbance at 690 was used as the reference of wavelength. BMMSCs cultured in a complete culture medium were used as controls [21].

### Quantitative real-time polymerase chain reaction (RT-qPCR)

BMMSCs in the 3rd passage were cultured in a T-75 flask under different concentrations of the tested hydrogels for 3 weeks. BMMSCs were homogenized and the RNA was extracted with Direct-zol RNA miniprep plus (Cat# R2072, zymo research corp. US) then quality and quantity were measured using beckman dual spectrophotometer (DU-640, Trusted laboratories, US). Super Script IV One-Step RT-qPCR kit (Cat# 12594100, Thermo Fisher Scientific, Waltham, US) was used for reverse transcription of extracted RNA followed by PCR. 96-well plate step one instrument (Applied Biosystem, US) was utilized in a thermal profile as follows: 10 min at 45 ºC for reverse transcription, 2 min at 98 ºC for RT inactivation and initial denaturation by 40 cycles of 10 sec at 98ºC, 10 sec at 55 ºC and 30 sec at 72 ºC for the amplification step. After the RT-qPCR, data were expressed in cycle threshold (Ct) for the target genes and housekeeping genes. Normalization for variation in the expression of each target gene; COL-I was done referring to the mean critical threshold (CT) expression value of the glyceraldehyde-3-phosphate dehydrogenase (GAPDH) housekeeping gene by the ΔΔCt method. The relative quantitation (RQ) of each target gene is quantified according to the calculation of the 2-∆∆Ct method. The primer sequence was forward 5′- GTACATCAGCCCAAACCCCAAG − 3′ and reverse 5′- CGGAACCTTCGCTTCCATACTC − 3′ (Gene bank accession number: XM_032912698.1) for COL-I gene, and GAPDH housekeeping gene was; forward 5’- CACCCTGTTGCTGTAGCCATATTC − 3’ and reverse 5’-GACATCAAGAAGGTGGTGAAGCAG-3’ (Gene bank accession number: XM_017592435.1). All the data were expressed as relative to the control group in which BMMSCs were seeded in a complete culture medium.

### Alkaline phosphatase activity

ALP was measured by ELISA on different concentrations of the tested hydrogels on the 7th, 10th, 14th and 21st days of culture with an initial seeding density of BMMSCs equal to 5000 cells/cm2 at 3rd passage in 24-well plates. ELISA was performed (based on manufacturer’s guidelines) on the cell supernatant to determine the concentrations of rat ALP (ELISA Kit Cat No. SEB091Ra, Cloud-clone corporation, Wuhan, Hubei, China). The supernatant was analysed in triplicate and the absorbance was measured at 450 nm and Abs 690 (as the reference of wavelength). The quantities of factors (ng/ml) were calculated based on a standard curve.

### Statistical analysis

Data were presented as means ± standard deviations (SDs) from the 3 independent experiments. SPSS 24.0 software (SPSS Inc., Chicago, US) was utilized for data analyses using one-way ANOVA. A *P* value < 0.05 was considered statistically significant.

## Results

### Characterization of BMMSCs

**Fig. 1:**
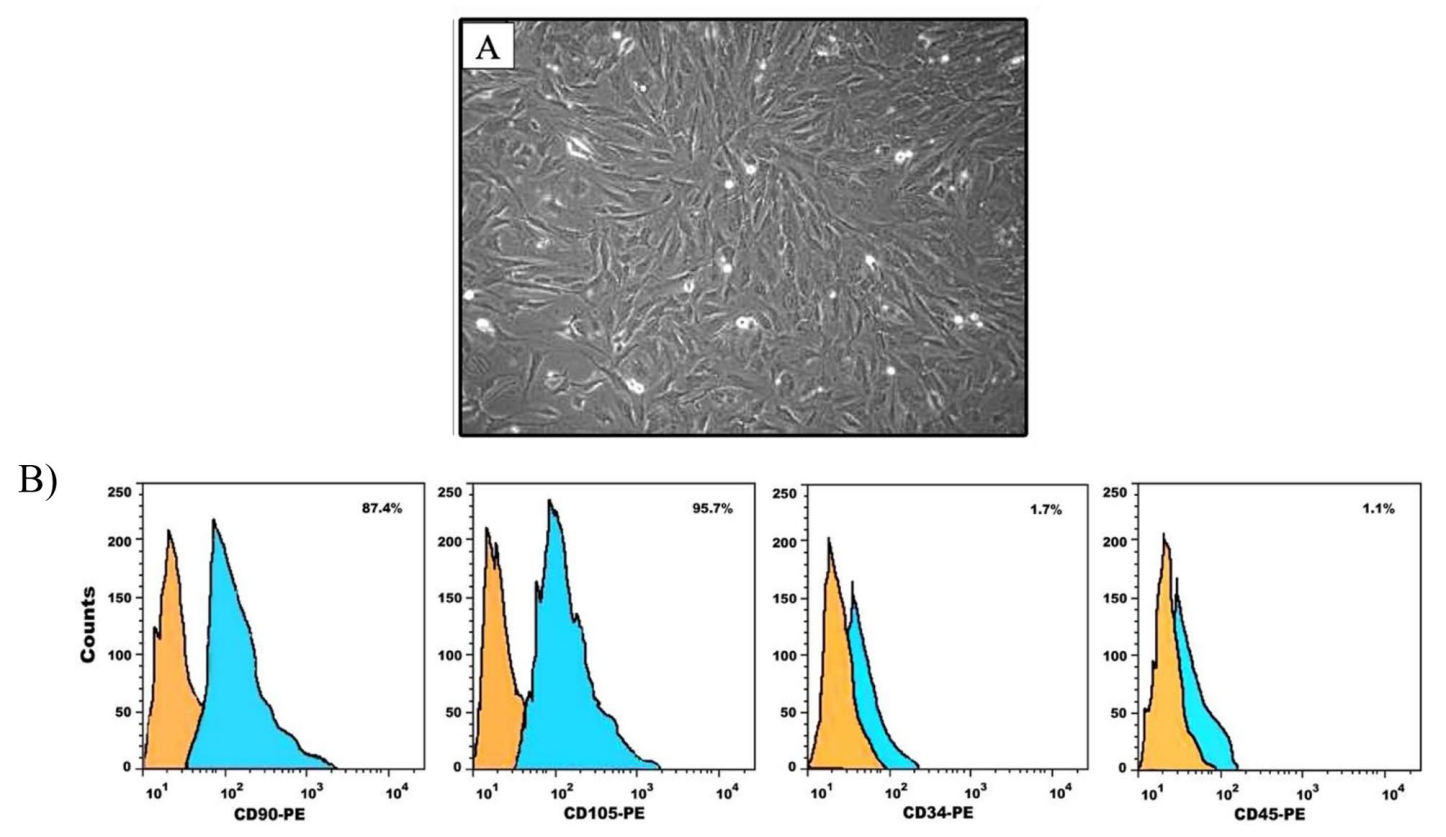
**(A)** Phase contrast photomicrograph showing confluent BMMSCs with spindle-shaped morphology after 14 days of culture. **(B)** Mesenchymal phenotype analysis of BMMSCs using flow cytometry. Each histogram displays fluorescence intensity as a measurement parameter on the x-axis and the cell count on the y-axis

Cells were identified as MSCs based on their morphology. Isolated BMMSCs had a spindle-shaped morphology after the third passage **(Fig. 1A)**. Flow cytometric analysis of the immunophenotypic characteristics of the BMMSCs revealed that the isolated cells were positive for CD90 and CD105, but negative for CD34 and CD45, which confirmed the stemness of the MSCs. (**Fig.** 1B).

### Characterization of the formulated hydrogels

#### Morphological characteristics and microstructure of hydrogels

**Fig. 2:**
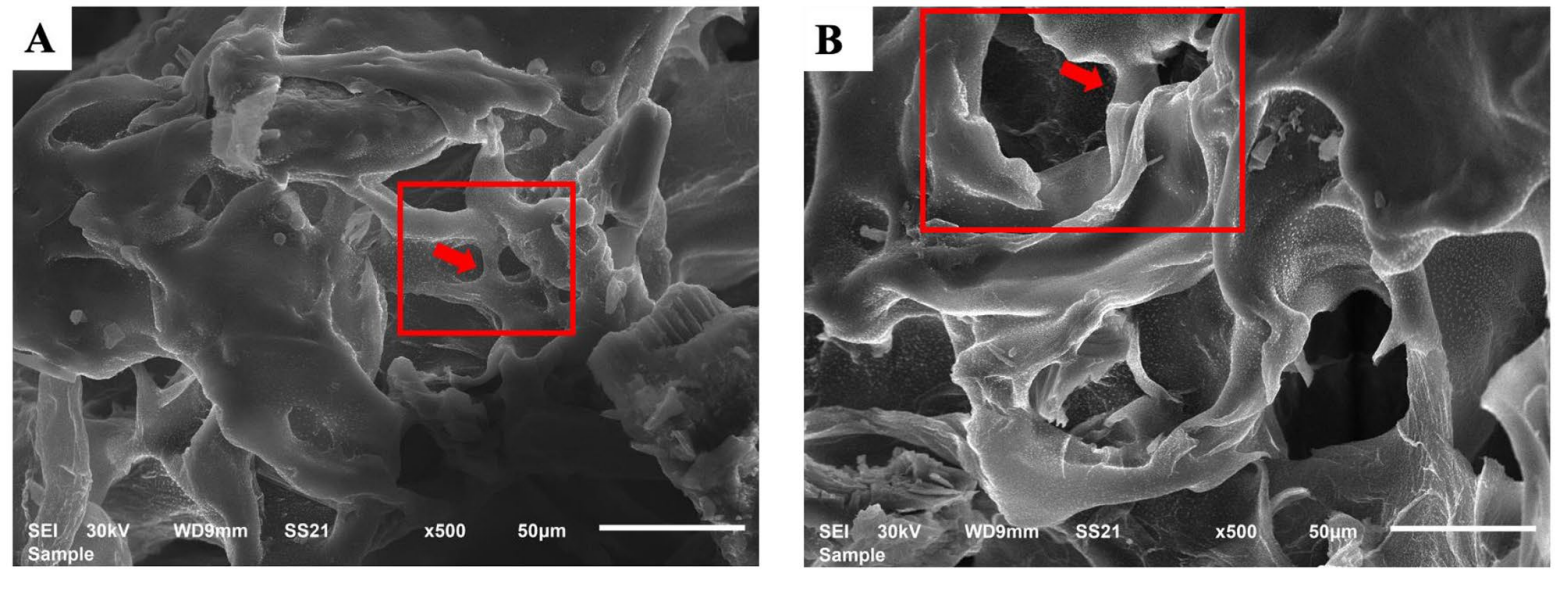
SEM micrographs for **(A)** DDMH hydrogel and **(B)** NHH hydrogel showing interconnected pores **(Magnification x500, Bar 50 μm)**

SEM micrographs of the scaffolds cross-sections were obtained to view the structure of the formulated hydrogels. The results showed that the formulated hydrogels have a porous microstructure with interconnected pores (Fig. 2).

### Zeta potential – charge surface

The DLS measurements of n-HA and DDM were carried out in deionized water between − 100 and + 100 mV. The n-HA has a negative surface charge with a maximum peak at − 6.81 ± 1.58 mV with an average size of 50.3 ± 53.80 nm and a polydispersity index (PDI) of 0.687. While the DDM has a negative surface charge with a maximum peak at -10.9 ± 0.907 with an average size of 300.7 ± 31.00 μm and PDI of 0.415.

### Cell viability

**Fig. 3:**
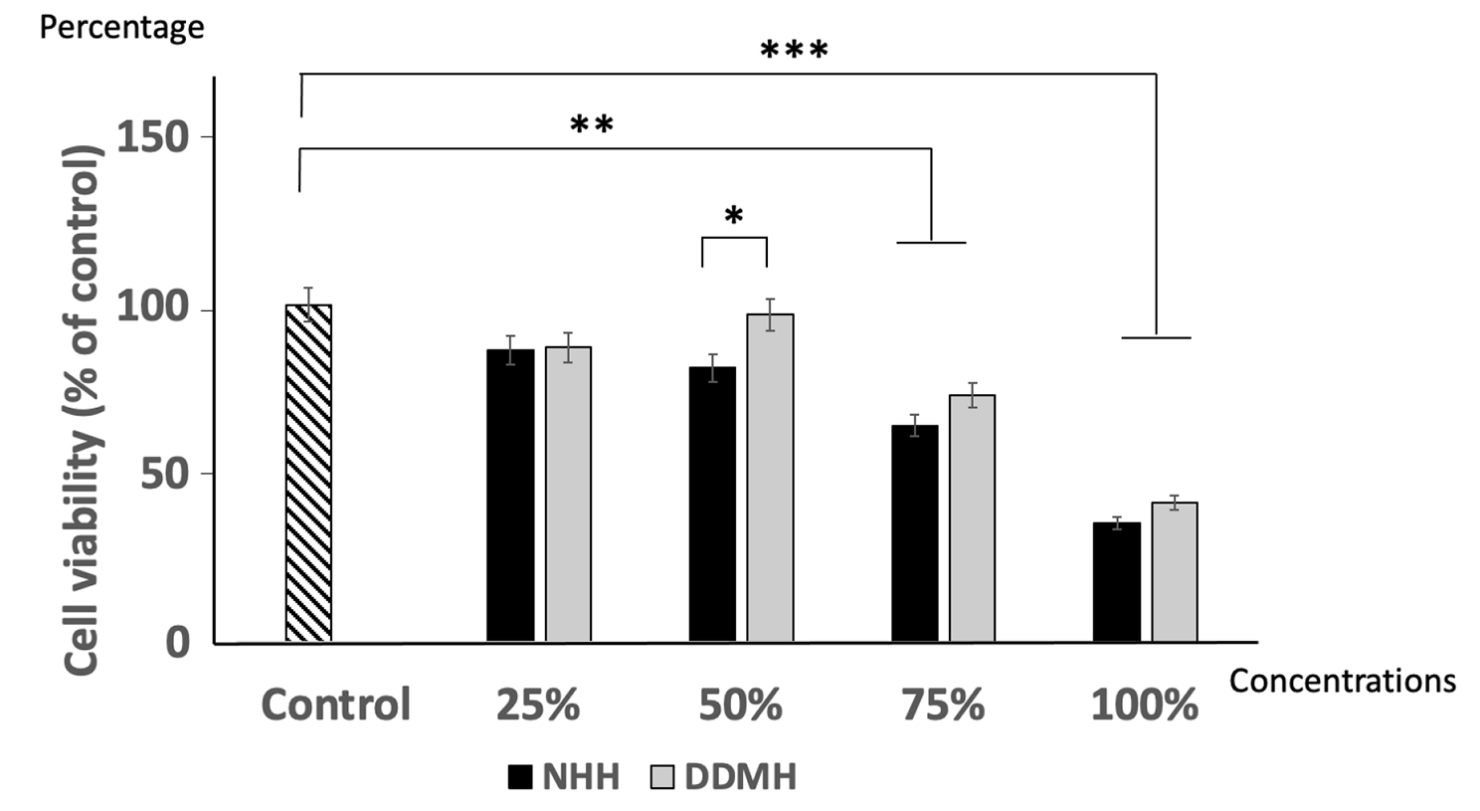
MTT analysis of viable BMMSCs percentage cultured with either DDMH or NHH. BMMSCs were exposed to complete medium (control), 25%, 50%, 75% and 100% DDMH or NHH for 72 h. Data are expressed as mean ± SD; n = 3. * *P < 0.05*, ** *P < 0.01*, *** *P < 0.001*

MTT assay was utilized for testing the metabolic activity of BMMSCs seeded with different concentrations of tested hydrogels for 72 h (**Fig.** 3). The viability of BMMSCs in 75% and neat/100% concentrations of the DDMH and NHH were significantly less than controls (*P* < 0.01, *P* < 0.001respectively).

Suggesting that 75% and neat/100% concentrations were cytotoxic to BMMSCs. However, at a concentration of 25% and 50% of either DDMH or NHH, the cell viability was not significantly different (*P* > 0.05) compared to controls. Noteworthy that the percentage of viable cells exposed to 50% of DDMH showed a significant increase compared to 50% of NHH (*P* < 0.05).

### Impact of tested hydrogel materials on COL-I gene expression in BMMSCs

**Fig. 4:**
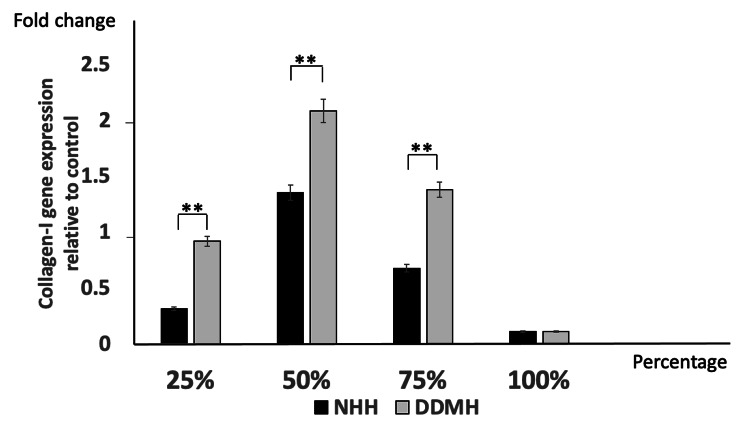
RT-qPCR analysis of COL-I gene expression in BMMSCs incubated with either 25%, 50%, 75% and 100% of DDMH or NHH for 3 weeks. Data are presented as mean ± SD relative to control. The housekeeping gene is GAPDH; n = 3. ** *P < 0.01*

RT-qPCR was done to measure COL-I gene expression in BMMSCs incubated with different concentrations of tested hydrogels for 3 weeks. BMMSCs seeded in 25%, 50%, and 75% DDMH showed significant upregulation of COL-I gene expression compared to those cultured with the same concentrations of NHH. These results suggest that COL-I gene expression, which accounts for a large proportion in the bone matrix and one of the early osteogenic differentiation markers, was significantly upregulated in BMMSCs cultured, with DDMH being higher in 50% hydrogel concentration than other treated groups. It has been noticed that COL-I gene expression was significantly downregulated in the cells cultured with neat/100% concentrations of either DDMH or NHH (Fig. 4).

### Alkaline phosphatase activity in BMMSCs cultured in the tested hydrogels

**Fig. 5:**
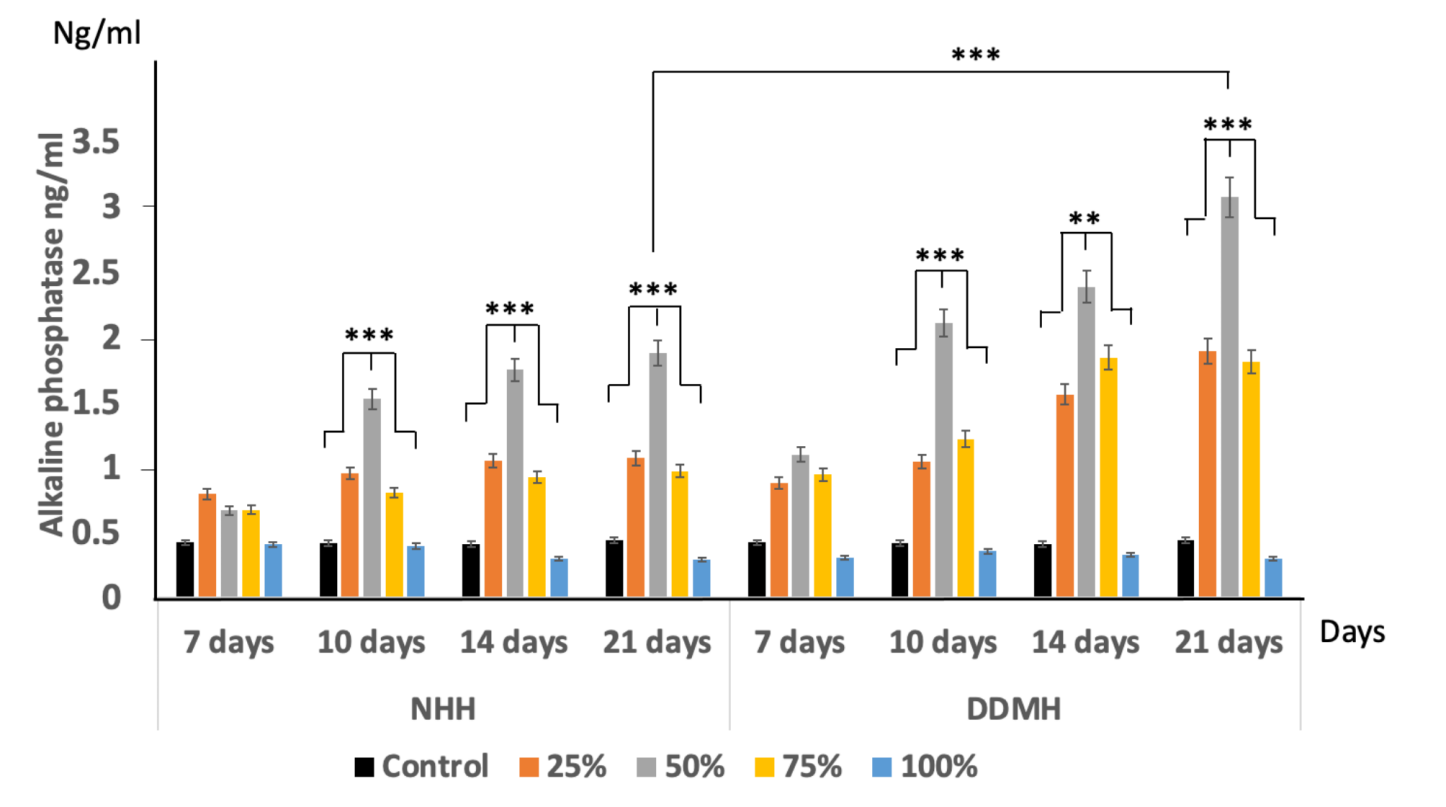
Amount of ALP expression in BMMSCs cultured with either; 25%, 50%, 75% and 100% concentrations of NHH and DDMH over 7, 10, 14 and 21 days. BMMSCs cultured in a complete culture medium were considered as the control group. Data are expressed as mean ± SD, n = 3, ** P < 0.01, ***P < 0.001

On day 7, the ALP activity in the cells cultured with either DDMH or NHH showed a slight increase in all the tested concentrations without being statistically significant with controls. On 10th, 14th and 21st days in culture, ALP significantly increased in groups cultured with either 50% DDMH or 50% NHH compared to all other concentrations (*P* < 0.001). Additionally, the amount of ALP enzymatic reaction from BMMSCs cultured with 50% DDMH (3.08 ng/ml) was significantly higher than 50% NHH (1.89 ng/ml) after 21 days of culture suggesting that 50% concentration of DDMH was more potent than 50% NHH in stimulating ALP activity (Fig. 5).

## Discussion

Dental tissues have long been considered as a promising functional and biocompatible alternative to current materials in regenerative dentistry. New techniques and technologies for dentin and bone regeneration have been made possible by recent advancements in tissue engineering. Similar to the bone, dentin contains a plethora of bioactive components such as growth factors, inflammatory molecules and extracellular matrix proteins. Each of these molecules has its role in the tissue repair response [22]. Sequestration of factors in the dentin matrix acts as a reservoir of signalling molecules [23]. Carious injury to dentin causes demineralization and this is followed by a release of sequestered factors which contribute to reparative dentinogenesis [24]. Clinically induced release of bioactive constituents from dentin utilizing EDTA, offers effective-pulp capping, through the induction of reparative responses of the dentin-pulp complex [25]. There are many reasons for dental extraction, including caries, mobility, orthodontic tooth reasons, and trauma. Thus, DDM can be prepared with low risks of infection and rejection and non-invasive attainability, making it a natural resource that should be used to its full potential for other applications [26].

International society for cell therapy (ISCT) established a set of minimal characteristics to define MSCs. According to their criteria, these cells must express CD73, CD90 and CD105 surface markers and the absence of hematopoietic cell surface markers CD45, CD34, CD14, CD11b, CD79a or CD19. In this study, BMMSCs were successfully characterized by flowcytometric positive expression of CD90 and CD105, and negative expression for CD34 and CD45 [27].The successful osteogenic differentiation of BMMSCs is important for appropriate bone regeneration. If such differentiation does not occur, BMMSCs implanted in the body will fail to repair bone defects effectively with the risk of being differentiated into other tissue types [28].

In this study, negative values of zeta potential possess significant positive effects on the attachment and proliferation of bone cells, along with direct bone bonding and osteogenesis which may also be a significant factor influencing their biological behaviors [24, 29]. The dentinal tubule would widen once dentin is demineralized and function as a route for delivering necessary proteins, which may encourage cell development and differentiation. Moreover, the exposed collagen fibres after demineralization are advantageous for cell adhesion [30]. In this study, dentin was partially demineralized by being exposing to EDTA for 20 min, which is considered partial demineralization [18, 19]. Partially demineralized dentin possessed a suitable surface for cell attachment and provide a balance between its resorption and bone formation on it [10].

Tissue engineering strategies depend mainly upon a scaffold involving osteoconductive and osteoinductive properties [31]. In this study, the formulated hydrogels showed a porous microstructure which is suggested to be in favour for cell attachment, growth, and differentiation and will hasten bone regeneration. DDM has been proven to be biocompatible with osteoconductive behaviour [32]. However, the optimum hydrogel concentration of DDM efficacy on the viability and osteogenic ability of BMMSCs has not yet been assessed.

Cytotoxicity is a principal issue when evaluating the biocompatibility of certain materials. Therefore, it was essential to assess DDMH cytotoxicity in comparison to the widely used NHH on BMMSCs. Interestingly, after 72 h incubation in DDMH, BMMSCs viability was preserved and even increased in the 25% and 50% concentration groups, signifying the presence of effector constituents in the DDMH. When DDMH concentration reached 100%, they showed cytotoxicity to the BMMSCs. These results are in the same line with other in vitro studies, which concluded that DDM was biocompatible with BMMSCs and increased their proliferation and differentiation potentials [33, 34].

To evaluate the osteogenic differentiation of stem cells, the COL-I expression was performed. COL-I, an extracellular matrix protein, has been broadly shown to promote the proliferation, survival, adhesion and osteogenesis of BMMSCs [35]. A previous histological study revealed that DDM contained several growth factors, such as COL-I and BMP in the dentin, and therefore exhibited osteoinductive and osteoconductive capacities [36]. In this study, it was found that the group of cells cultured with DDMH showed a 1-fold improvement in COL-I expression at 25% concentration and a double increase at 50% concentration. The existence of additional differentiated osteoblasts in the DDMH-treated groups and the upregulation of this gene in the DDMH groups indicate that this material can accelerate bone formation compared to NHH. A study done by Avery et al. stated that DDMH induced the formation of mineralized nodules, which was confirmed by enhanced gene expression of COL-I and confirming the osteogenic potential of DDM [37]. It has been noticed that COL-I gene expression was significantly downregulated in the cells cultured with neat/100% concentrations of either DDMH or NHH and this probably be due to the marked reduction in the viable cell numbers in those treated groups.

Additionally, ALP level, which is an early marker of osteoblasts differentiation, was measured over the 7th, 10th, 14th and 21st days. The results showed that ALP level gradually increased over the culture time in all treated groups except in neat/100% hydrogel-treated groups which showed nearly the same level over the time studied. The results demonstrated that DDMH had a booster effect on cell differentiation increasing the ALP level in the cultured cells, indicating an additional transformation from BMMSCs to osteoblasts. Noteworthy that 50% hydrogel concentration in either NHH or DDMH resulted in a significant increase in ALP level in comparison to other concentrations used over 10th, 14th and 21st days of culture. On day 21st of culture, the expression of ALP was quite distinctive. The data demonstrated that ALP level in the group treated with 50% DDMH was significantly higher than the group treated with 50% NHH.

To sum up, the groups treated with DDMH had better osteogenic potential of BMMSCs than the NHH treated groups by significantly increased the ALP expression levels over the time studied. However, NHH-treated groups showed upregulation in COL-I mRNA levels but was significantly less than DDMH treated groups. This could be attributed to those growth factors already existing in DDM making DDMH superior to NHH and the synergism of these growth factors could have a positive effect [38]. However, further studies are needed to assess mRNA levels of more osteoblasts specific genes named osteopontin, RUNX-2, bone sialoprotein and bone morphogenic proteins to further validate these results.

## Conclusion

Nowadays, many researches have envisioned the potential usage of DM as a graft for bone regeneration and replacement due to the wide availability of teeth and the similarity between the tooth-derived materials and the bone in the physical and chemical properties. In our study, DDM was successfully delivered using hydrogel formulation that can be injectable and has gained significant traction in biomedical applications. The partial demineralization process used for dentin allowed preserving the organic content increasing the biocompatibility. In the light of this, the ability to adjust the level of demineralization creates new opportunities for the creation of more potent osteoinductive materials for bone regeneration.

## Data Availability

The datasets used during the current study are available from the corresponding author upon request.
